# Comprehensive analysis of a ceRNA network reveals potential prognostic cytoplasmic lncRNAs involved in HCC progression

**DOI:** 10.1002/jcp.28522

**Published:** 2019-03-27

**Authors:** Yi Bai, Junyu Long, Zhisong Liu, Jianzhen Lin, Hanchan Huang, Dongxu Wang, Xu Yang, Fei Miao, Yilei Mao, Xinting Sang, Haitao Zhao

**Affiliations:** ^1^ Department of Liver Surgery Peking Union Medical College Hospital, Chinese Academy of Medical Sciences & Peking Union Medical College (CAMS & PUMC) Beijing China; ^2^ Department of Statistics Tianjin University of Finance and Economics Pearl River College Tianjin China

**Keywords:** ceRNA, hepatocellular carcinoma, lncRNA, overall survival, risk score

## Abstract

The aberrant expression of long noncoding RNAs (lncRNAs) has drawn increasing attention in the field of hepatocellular carcinoma (HCC) biology. In the present study, we obtained the expression profiles of lncRNAs, microRNAs (miRNAs), and messenger RNAs (mRNAs) in 371 HCC tissues and 50 normal tissues from The Cancer Genome Atlas (TCGA) and identified hepatocarcinogenesis‐specific differentially expressed genes (DEGs, log fold change ≥ 2, FDR < 0.01), including 753 lncRNAs, 97 miRNAs, and 1,535 mRNAs. Because the specific functions of lncRNAs are closely related to their intracellular localizations and because the cytoplasm is the main location for competitive endogenous RNA (ceRNA) action, we analyzed not only the interactions among these DEGs but also the distributions of lncRNAs (cytoplasmic, nuclear or both). Then, an HCC‐associated deregulated ceRNA network consisting of 37 lncRNAs, 10 miRNAs, and 26 mRNAs was constructed after excluding those lncRNAs located only in the nucleus. Survival analysis of this network demonstrated that 15 lncRNAs, 3 miRNAs, and 16 mRNAs were significantly correlated with the overall survival of HCC patients (*p* < 0.01). Through multivariate Cox regression and lasso analysis, a risk score system based on 13 lncRNAs was constructed, which showed good discrimination and predictive ability for HCC patient survival time. This ceRNA network‐construction approach, based on lncRNA distribution, not only narrowed the scope of target lncRNAs but also provided specific candidate molecular biomarkers for evaluating the prognosis of HCC, which will help expand our understanding of the ceRNA mechanisms involved in the early development of HCC.

## 1 INTRODUCTION

Liver cancer is the sixth most common type of malignant tumor in the world and is currently the second most common cause of tumor‐related death (Bray et al., 2018). According to 2018 epidemiological data from the United States, the mortality of patients with liver cancer increased by 2.7% per year for women and 1.6% per year for men from 2011 to 2015 (Siegel, Miller, & Jemal, 2018). Given the lack of specific clinical manifestations of early hepatocellular carcinoma (HCC) in patients, 70–80% of patients are in advanced stages when they present symptoms and miss the opportunity to receive radical resection (C. Li, Li, & Zhang, 2018). In addition, although there has been great progress in the development of treatment approaches for HCC, including radiotherapy, chemotherapy, transcatheter arterial chemoembolization (TACE) therapy, radiofrequency ablation, targeted therapy, and immunotherapy, the overall 5‐year survival rate of HCC patients after curative intent surgical treatment has been reduced by only 1–3%, and the 5‐year recurrence rate postoperation can reach 70%. Notably, the median survival time of patients with advanced liver cancer who do not receive treatment is only 7.1 months (Kulik & El‐Serag, 2019). Therefore, identifying the molecular mechanisms underlying the initiation, development and metastasis of HCC is essential for early diagnosis, the selection of therapeutic approaches, the determination of follow‐up schedules, and the assessment of prognosis to help increase patient life expectancy and clinical benefits.

As types of noncoding RNA (ncRNA) without protein coding ability, long noncoding RNAs (lncRNAs) were originally considered transcriptional noise (Quinn & Chang, 2016). However, accumulating evidence has demonstrated that the differential expression of lncRNAs plays pivotal roles in hepatocarcinogenesis, vascular invasion and distant metastasis through dose compensation, epigenetic regulation, cell cycle regulation and cell differentiation regulation (He et al., 2014; Schmitt & Chang, 2016). lncRNAs are usually more than 200 nucleotides in length and exhibit greater species, tissue, and cell specificity than do shorter‐length microRNAs (miRNAs) and messenger RNAs (mRNAs) due to their evolutionary unconserved characteristics. In addition, lncRNAs perform different regulatory functions based on their subcellular localizations. In general, in the nucleus, lncRNAs mainly function in chromatin regulation, transcriptional regulation, and variable splicing regulation. In the cytoplasm, lncRNAs affect mRNA stability and translational regulation, largely through the competitive endogenous RNA (ceRNA) regulation mechanism of adsorbed miRNA (Cao, Pan, Yang, Huang, & Shen, 2018).

The ceRNA hypothesis was first proposed by Salmena and colleagues in 2011 (Salmena, Poliseno, Tay, Kats, & Pandolfi, 2011). In the ceRNA gene interaction network, which includes lncRNAs, miRNAs, and mRNA, lncRNAs can act as endogenous molecular sponges that competitively bind miRNAs via shared microRNA response elements with reverse complementary binding seed regions to indirectly regulate mRNA expression levels. In recent years, numerous experiments have validated the hypothesis that this type of indirect regulatory mechanism is involved in carcinoma initiation, progression, and invasion. For example, DSCR8 promotes HCC cell progression by sponging miR‐485‐5p to activate frizzled‐7, which is associated with the Wnt/β‐catenin srignaling pathway (Y. Wang, Sun, et al., 2018). HOXD‐AS1 can prevent SOX4 from undergoing miRNA‐mediated degradation via binding miR‐130a‐3p, thereby promoting HCC metastasis (H. Wang, Huo, et al., 2017). However, integrated and comprehensive analyses of the regulatory functions of the lncRNA–miRNA–mRNA ceRNA network in tumor pathogenesis have been hindered by a lack of available databases and research approaches. The Cancer Genome Atlas (TCGA) platform is an open‐source sequencing database covering more than 30 human cancer types and contains information on clinical pathology and corresponding bioinformatics data (Hutter & Zenklusen, 2018). This database is an ideal resource for biological discovery and data mining. ceRNA networks have been constructed for most tumor types, such as head and neck squamous cell carcinoma (HNSCC; Fang et al., 2018), gastric cancer (GC; C. Y. Li et al., 2016), and cutaneous melanoma (Xu et al., 2018). These networks are useful for gaining insight into complicated gene interactions and for identifying potential biomarkers for cancer diagnosis, treatment, and prognosis.

In the current study, in a first step, we compared differentially expressed lncRNAs, miRNAs, and mRNAs between well/moderately differentiated (G1/G2) tissues and normal tissues and differentially expressed genes (DEGs) between poorly differentiated (G3 and G4) and normal tissues. Subsequently, the DEGs intersecting with 753 lncRNAs, 97 miRNAs, and 1,535 mRNAs were identified as candidate genes to construct a ceRNA regulatory network for HCC. Then, the locations and putative interactions of the lncRNAs among lncRNA–miRNAs–mRNAs were determined based on the miRcode, TargetScan, miRDB, and miRTarBase databases. Thirty‐seven lncRNAs, 10 miRNAs, and 26 mRNAs were selected to build the ceRNA network associated with HCC occurrence. Finally, 13 lncRNAs significantly affecting HCC patient prognosis were used to develop a risk score system after lasso‐penalized Cox regression analysis. This novel ceRNA network‐construction method, which considers lncRNA distribution, might aid in the screening of significant genes in HCC‐associated ceRNAs with a narrower scope and higher accuracy.

## 2 MATERIALS AND METHODS

### 2.1 Data retrieval and processing

The RNA sequence data (lncRNA and mRNA, level 3; Illumina HiSeq RNA‐Seq platform), miRNA sequence data (Illumina HiSeq miRNA‐Seq platform), and clinical information of liver hepatocellular carcinoma (LIHC) patients were manually downloaded from the TCGA data portal (https://portal.gdc.cancer.gov/). Using human genecode (https://www.gencodegenes.org/), we transformed the RNA sequence data into lncRNAs (sense overlapping, lncRNAs, 3′ overlapping ncRNAs, processed transcripts, antisense, and sense intronic) and mRNAs (protein coding). The LIHC cohort contained 371 tumor samples and 50 normal samples. Because the data were extracted from TCGA and because this study strictly followed the publication guidelines approved by TCGA (https://cancergenome.nih.gov/publications/publicationguidelines), there was no requirement for ethics committee approval.

### 2.2 Identification of DEGs

For the normalized gene expression profile data, we used the edgeR package of R software to analyze significantly aberrantly expressed lncRNAs, miRNAs, and mRNAs at two levels: moderately to well differentiated (G1‐G2 stage) HCC samples versus normal samples and poorly differentiated (G3‐G4 stage) HCC samples versus normal samples (Robinson, McCarthy, & Smyth, 2010). We selected a log fold change ≥ 2 and FDR<0.01 as significant cutoff values based on the Benjamini‐Hochberg method (Madar & Batista, 2016). Then, the differentially expressed lncRNAs, miRNAs, and mRNAs meeting the criteria were displayed in volcano plots. We generated Venn diagrams to visualize the intersecting DEGs between the results of the two comparisons for further analysis.

### 2.3 Construction of the ceRNA network

The ability of the lncRNAs to sequester and bind miRNAs was predicted using the miRcode database (http://www.mircode.org/; Jeggari, Marks, & Larsson, 2012). The target mRNAs of miRNAs were retrieved from the miRDB (Wong & Wang, 2015), miRTarBase (Chou et al., 2016), and TargetScan (Fromm et al., 2015) databases. To increase the reliability of the results, only those miRNA‐mRNA relationship pairs found in all 3 databases were selected as candidate genes for constructing the ceRNA network. Because lncRNAs can function as nodes of the ceRNA network only in the cytoplasm, we investigated the intracellular localization of the lncRNAs via the lncATLAS database (http://lncatlas.crg.eu/; Mas‐Ponte et al., 2017). lncATLAS is an easy‐to‐use web‐based visualization tool for obtaining information about the expression localization of GENCODE‐annotated lncRNAs. Finally, the ceRNA network based on interactions between cytoplasmic DElncRNAs and DEmiRNAs and between DEmiRNAs and DEmRNAs was constructed to reveal the gene interaction profile in HCC. Cytoscape (http://www.cytoscape.org/) software was used to visualize the expression locations of the lncRNAs and the ceRNA network (Shannon et al., 2003).

### 2.4 Function and pathway analyses of DEmRNAs

The Database for Annotation, Visualization, and Integrated Discovery (DAVID) online functional annotation tool (https://david.ncifcrf.gov/) was used to analyze the Gene Ontology (GO) molecular function enrichment of the differentially expressed, intersecting mRNAs, as described previously (Huang da, Sherman, & Lempicki, 2009). The KO‐Based Annotation System (KOBAS) online tool (http://kobas.cbi.pku.edu.cn/index.php) was used to analyze the Kyoto Encyclopedia of Genes and Genomes (KEGG) pathway enrichment of DEmRNAs (Xie et al., 2011). *P*<0.05 was considered to indicate statistical significance.

### 2.5 Survival analysis

Kaplan–Meier (K–M) survival analyses of the intersecting DElncRNAs, DEmiRNAs, and DEmRNAs in the ceRNA network were performed using the survival package in R. The optimal cutoff value was calculated according to the X‐tile method (Camp, Dolled‐Filhart, & Rimm, 2004). *p*<0.01 was considered to indicate statistical significance.

### 2.6 Construction of the risk score system

DElncRNAs associated with HCC patient survival were analyzed through lasso‐penalized Cox regression to remove confounding factors and reduce the number of genes. A Cox model was initially generated by applying the penalized maximum likelihood method. Ten‐fold cross‐validation was used to derive the best lambda to minimize the mean cross‐validated error and predict the regression coefficients (*β*) of the multivariate Cox regression model. Finally, a prognosis risk score system based on 13 genes was established. Prognosis index (PI) = (β1 × expression level of AL359878.1) + (β2 × expression level of CRNDE) + (β3 × expression level of C10orf91) + (β4 × expression level of LINC00462) + (β5 × expression level of PART1) + (β6 × expression level of AL163952.1) + (β7 × expression level of AP002478.1) + (β8 ×expression level of CLLU1) + (β9 × expression level of TCL6) +(β10 ×expression level of HTR2A‐AS1) + (β11 × expression level of AC073352.1) + (β12 × expression level of MIR137HG) + (β13 ×  expression level of LINC00221). When the gene expression value exceeded the cutoff value, the expression level of the correlated gene was considered “1”, whereas when the expression value was less than or equal to the cutoff value, the expression level was considered “0.” According to optimal cutoff value, all 365 HCC patients were divided into low‐ and high‐risk groups. To estimate the distinguishing and predictive abilities of the risk score system, K–M survival curves and time‐dependent receiver operating characteristic (ROC) curves were constructed.

### 2.7 Univariate and multivariate cox regression analyses

To detect whether the clinical characteristics, including age, gender, body mass index (weight/height^2^), pathologic stage, histologic grade, alpha‐fetoprotein, inflammation extent, vascular invasion, and family history, were significantly associated with overall survival in HCC patients, univariate Cox regression analysis was performed. Risk score level, pathologic stage, and vascular invasion, as candidate variables, were included in the multivariate Cox regression analysis. *p*<0.05 was considered to indicate statistical significance. The hazard ratio and 95% confidence intervals for each variable were calculated.

### 2.8 Regression analysis of DElncRNAs and DEmRNAs

Regression analysis of the relative expression levels of DElncRNAs and DEmRNAs was performed and the results visualized using R software and the ggpubr, tidyverse, Hmisc, and corrplot packages. *p*<0.05 and *r* > 0.3 were considered statistically significant.

## 3 RESULTS

### 3.1 Aberrantly expressed lncRNAs, miRNAs, and mRNAs

**Figure 1:**
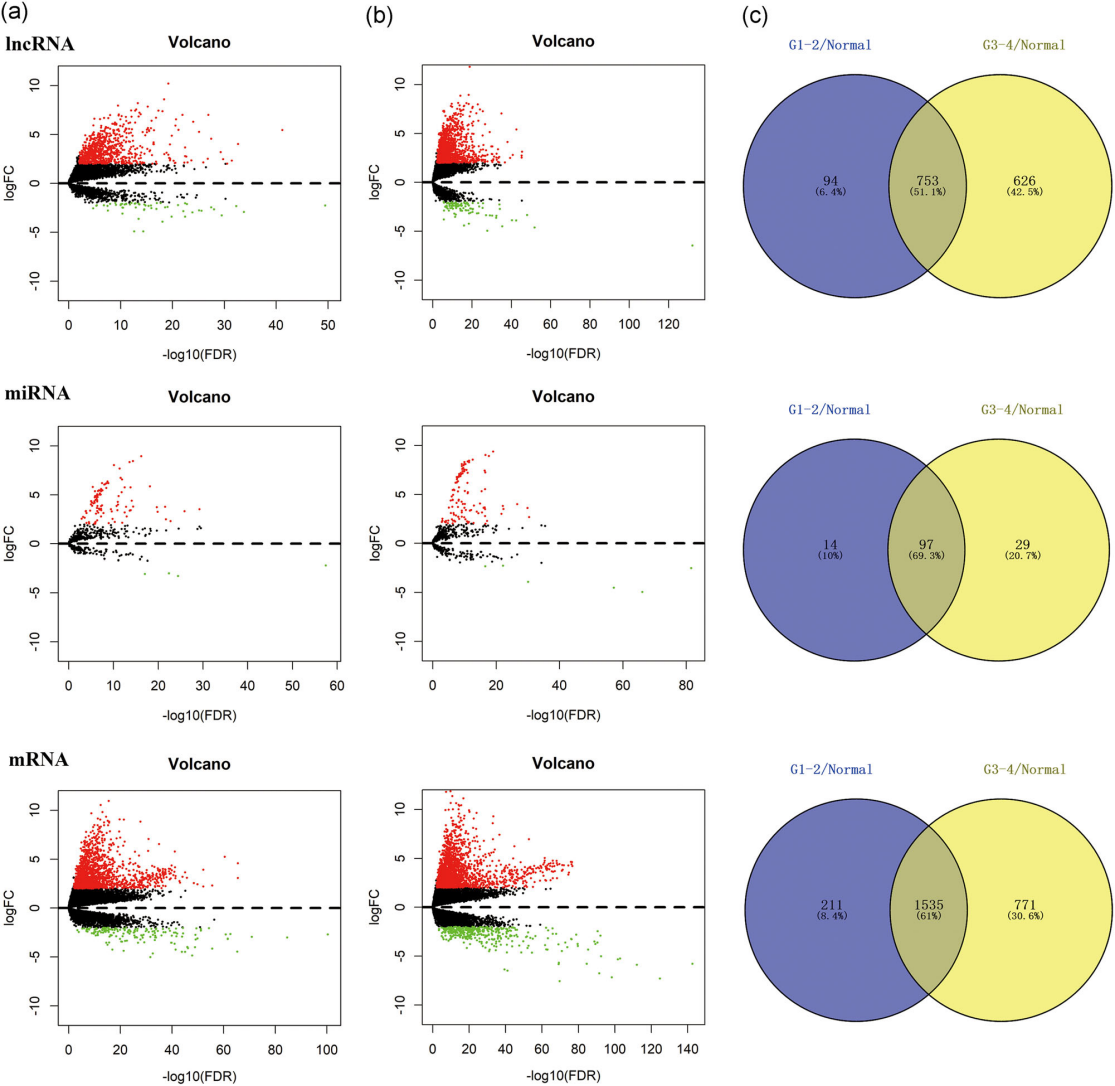
Identification of differential genes. Volcano maps of aberrantly expressed lncRNAs (above graph), miRNAs (medium graph), and mRNAs (below graph) between two groups: normal samples versus well and moderately differentiated (G1‐G2 stage) HCC samples (a); normal samples versus poorly differentiated (G3‐G4 stage) HCC samples (b). Red dots are defined as upregulated genes, and green dots are defined as downregulated genes. Venn diagrams represent the intersections of differentially expressed genes (c). The purple areas derive from left volcano maps, and the yellow areas derive from left volcano maps. FC: fold change; HCC: hepatocellular carcinoma; lncRNAs: long noncoding RNAs; miRNA: microRNA; mRNA: messenger RNA [Color figure can be viewed at wileyonlinelibrary.com]

With the progression of HCC, liver cancer cells may differentiate into distinct differentiation types or even different tumor subtypes. To better understand tumorigenesis‐associated DEGs, we initially divided the entire sample into three groups: one comprising 50 normal samples, one comprising 233 well or moderately differentiated (G1‐G2 stage) HCC samples, and one comprising 136 poorly differentiated (G3‐G4 stage) HCC samples. Then, as shown in Figure 1a, we compared the tumor groups with the normal group to visualize significantly differentially expressed lncRNAs, miRNAs, and mRNAs using volcano maps (log fold change ≥ 2, FDR < 0.01). The intersections of the two sets of DEGs were composed of 753 lncRNAs (709 upregulated, 44 downregulated), 97 miRNAs (93 upregulated, 4 downregulated), and 1,535 mRNAs (1,384 upregulated, 151 downregulated), which were considered key genes involved in early HCC occurrence (Figure 1b).

### 3.2 Prediction of lncRNAs targeted by miRNAs

**Figure 2:**
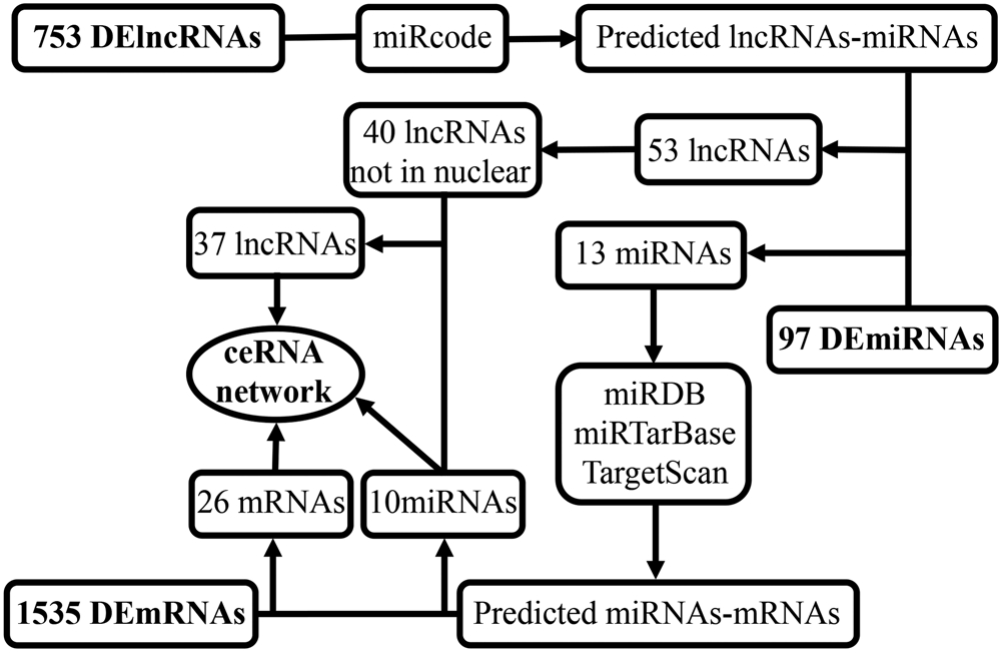
Flow chart of ceRNA regulatory network construction. ceRNA: competitive endogenous RNA; lncRNA: long noncoding RNA; miRDB: microRNA database; miRNA: microRNA; mRNA: messenger RNA

Figure 2 presents a flow chart of the creation of the ceRNA network. We first predicted potential miRNAs that interacted with 753 lncRNAs using the miRcode database. Then, the intersecting genes between the predicted miRNAs and 97 DEmiRNAs were obtained. Finally, we identified 53 lncRNAs and 13 miRNAs with mutual interaction ability (Table S1).

### 3.3 Prediction of mRNAs targeted by miRNAs

To improve the reliability of the bioinformatics prediction, we identified target genes of the above‐mentioned 13 miRNAs by selecting mRNAs shared by all three databases (miRDB, miRTarBase, and TargetScan). We then compared candidate target mRNAs with 1,535 differentially expressed mRNAs. Finally, miRNA–mRNA interaction pairs involving 10 miRNAs and 26 mRNAs were confirmed to establish the ceRNA network (Table S2).

### 3.4 Intracellular localization of lncRNAs and the ceRNA network

**Figure 3:**
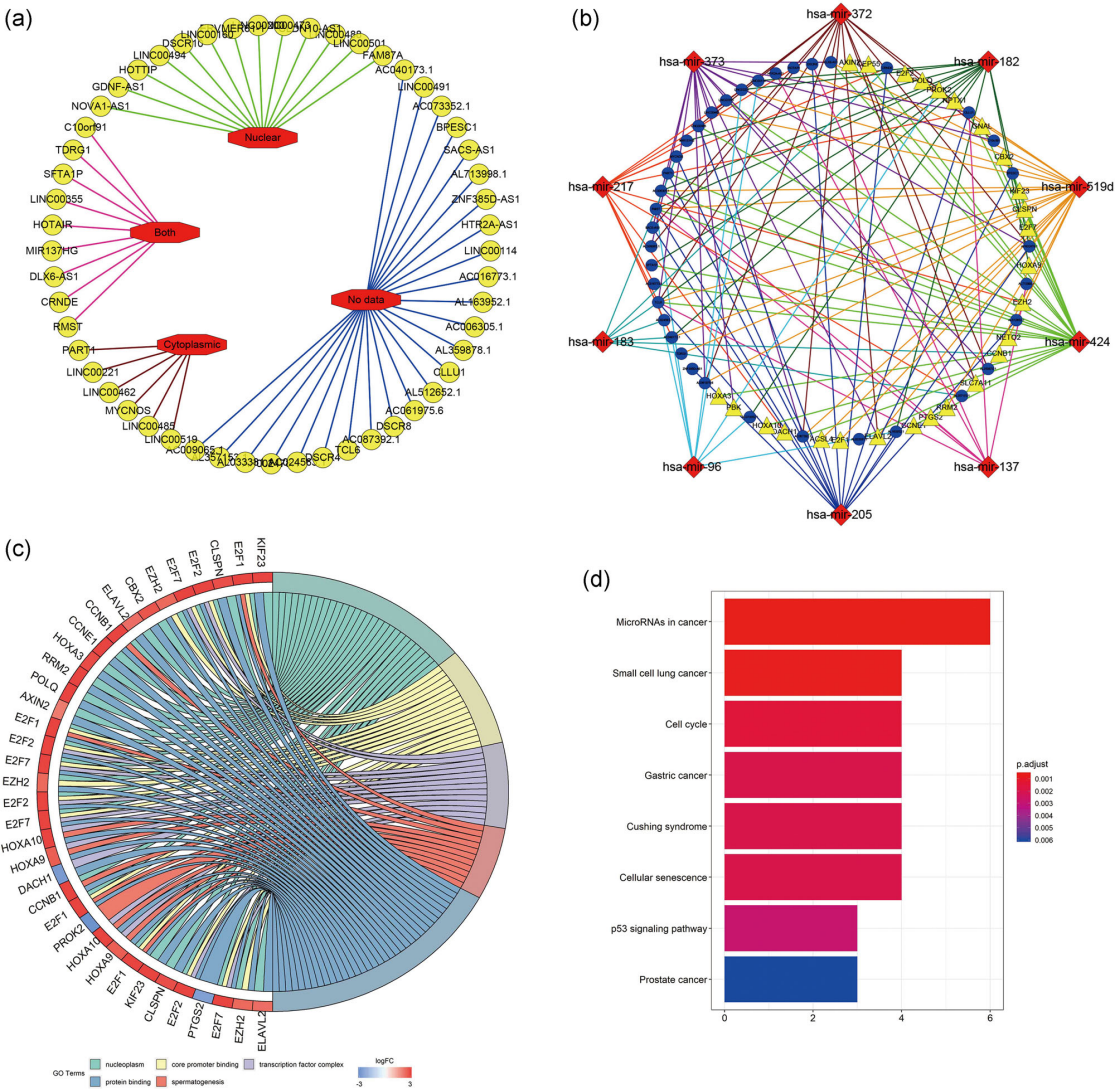
Integrated analysis of ceRNA network The subcellular localization of DElncRNAs (a). Red octagons stand for the intracellular distribution (nuclear; cytoplasm; both; no data) of 53 DElncRNAs (yellow circles). The ceRNA network derived from DEGs (b). Blue circles represent 37 lncRNAs; red diamonds represent 10 miRNAs; yellow triangles represent 26 mRNAs. Chord diagram displayed five significant enriched GO terms of 26 DEmRNAs (c). The GO terms are defined as indicated color bars at the bottom and shown on the right of chord diagram, the involved DEmRNAs are shown on the left. The red gene bars represent upregulated, and blue ones represent downregulated. The DEmRNAs associated eight statistically significant signaling pathways (d). The *x*‐axis indicates the number of DEmRNAs participating in the given pathway. ceRNA: competitive endogenous RNA; DEGs: differentially expressed genes; GO: Gene Ontology; lncRNA: long noncoding RNA; miRNA: microRNA; mRNA: messenger RNA [Color figure can be viewed at wileyonlinelibrary.com]

Inspecting the cytoplasmic‐nuclear localization of lncRNAs is a vital step in studying the complicated but precise regulatory mechanisms of these lncRNAs because the endogenous competition role of lncRNAs is mainly exhibited in the cytoplasm. Hence, we excluded 13 lncRNAs that were located only in the nucleus from the 53 DElncRNAs identified with the lncATLAS database. The distribution information for all of the differentially expressed lncRNAs was visualized with Cytoscape (Figure 3a; Table S3). After considering the interactions among the remaining DEGs, 37 DElncRNAs, 10 DEmiRNAs, and 26 DEmRNAs were incorporated into a final HCC ceRNA regulatory network composed of 73 nodes and 142 interactions (Figure 3b).

### 3.5 GO and KEGG enrichment analysis

Next, we studied the potential biological processes and pathways of the 26 DEmRNAs in the newly formed ceRNA network. Using the DAVID database, we performed GO functional enrichment analysis and identified 13 significant GO terms (*p*<0.01; Table S4). Among these terms, “nucleoplasm,” “core promoter binding,” “transcription factor complex,” “spermatogenesis,” and “protein binding,” in decreasing order of *p* value, were the top 5 GO terms. The relationships between the DEmRNAs and GO terms were visualized with Cytoscape software (Figure 3c). The KOBAS database was subsequently utilized to identify the KEGG pathway enrichment of the 26 DEmRNAs. Eight KEGG pathways were identified as statistically significant at *p* < 0.001, and the most significant pathway was “microRNAs in cancer” (Figure 3d).

### 3.6 Survival analysis of ceRNA network‐associated genes

**Figure 4:**
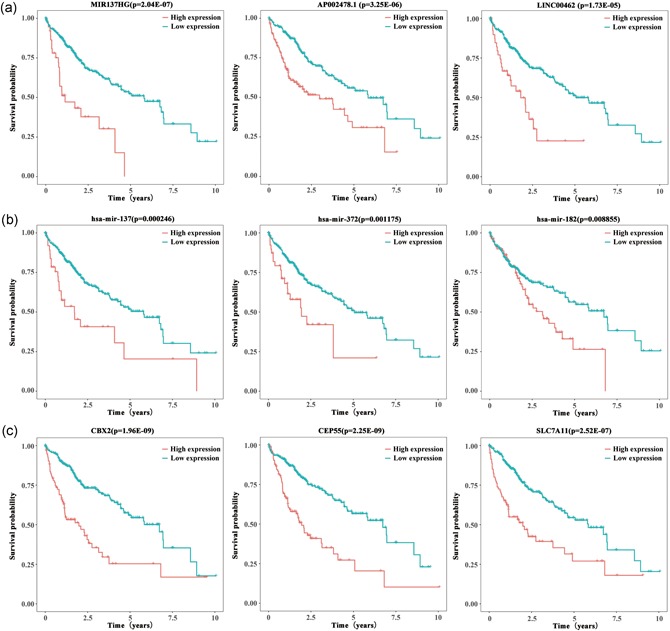
Kaplan–Meier survival analysis of DEGs in HCC patients. The top 3 most relevant to survival lncRNAs (a), miRNAs (b), and mRNAs (c) are shown based on their optimal cutoffs. DEGs: differentially expressed genes; HCC: hepatocellular carcinoma; lncRNA: long noncoding RNA; miRNA: microRNA; mRNA: messenger RNA [Color figure can be viewed at wileyonlinelibrary.com]

To identify the potential DEGs with strong correlations with the prognostic characteristics of patients with HCC, K–M survival analyses and log‐rank tests for each gene were performed to evaluate the contributions of 37 DElncRNAs, 10 DEmiRNAs, and 26 DEmRNAs. As a result, 13 lncRNAs, 3 miRNAs, and 15 mRNAs were identified as oncogenes because high expression levels of these RNAs were correlated with short survival time (*p*<0.01). Additionally, the expression levels of 2 lncRNAs, CLLU1 and HTR2A‐AS1, and the mRNA *PROK2* were positively correlated with the overall survival of patients with HCC (Table S5), suggesting protective roles of these RNAs in HCC development. K–M survival curves of the top 3 lncRNAs, miRNAs, and mRNAs, as ranked based on the association between expression level and the prognosis of HCC patient are shown in Figure 4a, b, and c, respectively.

### 3.7 Construction of the lncRNA‐associated risk score system

**Figure 5:**
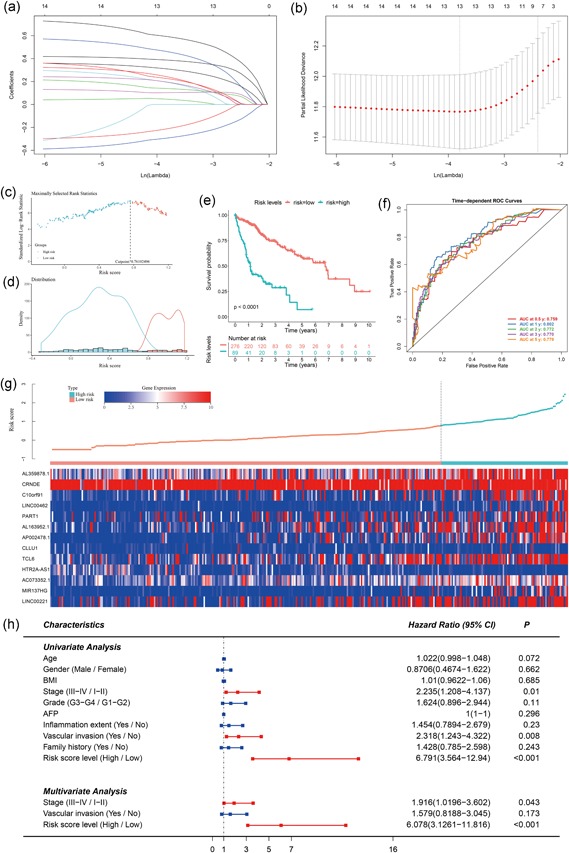
Risk score system. Lasso‐penalized Cox regression analysis of 15 DElncRNAs. The coefficient values at varying levels of penalty (a). Each curve represents an lncRNA. Ten‐fold cross‐validation was used to calculate best lambda which leads to minimum mean cross‐validated error (b). Red dots represent partial likelihood deviance; solid vertical lines indicate their corresponding 95% CI; the left dotted vertical line is the value of lambda that gives minimum cvm, named lambda. min; the right dotted vertical line is the largest value such that error is within 1 standard error of the minimum, named lambda. 1se. The selection of the optimal cutoff and survival curve based on risk score. Risk score‐related standardized log‐rank statistics was shown in (c). Maximally statistic was defined as the optimal cutoff value. Distribution of densities for low‐ and high‐risk score HCC patients was shown in (d). Kaplan–Meier survival curve of two groups were displayed in (e). Time‐dependent ROC curves based on risk score level were shown in (f). Risk score analysis of 13 DElncRNAs (g). The above scatter plot displays the risk score of 13 DElncRNAs, and the below heatmap exhibits the DElncRNA expression profiles in each HCC patients with survival data. Red is defined as high expression, and blue is defined as low expression. Univariate and multivariate analyses of clinical parameters associated with overall survival (h). The middle point represents the HR, and the length of the line represents the 95% confidence intervals for each indicator. Red represents statistical significance, and blue has no statistical significance. CI: confidence interval; HCC: hepatocellular carcinoma; HR: hazard ratio; lncRNA: long noncoding RNA; ROC: receiver operating characteristic [Color figure can be viewed at wileyonlinelibrary.com]

lncRNAs dominate the upstream portion of the ceRNA network and function as primary effectors of miRNAs and mRNAs. In addition, the expression and distribution of lncRNAs are highly specific, which makes them optimal biomarkers for HCC diagnosis and prognostic assessment. Hence, based on 15 lncRNAs that were significantly correlated with overall survival, lasso‐penalized Cox regression and multivariate Cox regression analyses were applied to select potential prognosis‐related lncRNAs, and their contributions were weighted by their relative coefficients (Figure 5a,b). Then, DSCR8 and AC006305.1 were excluded, and the final risk score formula was as follows: PI = (0.3093 × expression level of AL359878.1) + (0.2395 ×  expression level of CRNDE) + (0.0451 × expression level of C10orf91) + (0.4591 × expression level of LINC00462) + (0.1914 × expression level of PART1) + (0.1037 × expression level of AL163952.1) + (0.3858 × expression level of AP002478.1) + (− 0.2101 × expression level of CLLU1) + (0.1378 × expression level of TCL6) + (− 0.2911 × expression level of HTR2A‐AS1) + (0.1937 × expression level of AC073352.1) + (0.5917 × expression level of MIR137HG) + (0.2241 × expression level of LINC00221). Among these lncRNAs, CLLU1, and HTR2A‐AS1 had negative coefficients in the univariate and multivariate Cox regression analysis. This result indicated that these lncRNAs have protective roles, with high expression of these lncRNAs prolonging the OS of HCC patients. After estimating the maximally selected rank statistics (Figure 5c), the distribution of risk scores (Figure 5d), patients with risk scores greater than 0.7610 were classified into the high‐risk group (89 patients), whereas those with risk scores less than or equal to the cutoff value were allocated to the low‐risk group (276 patients). Notably, the designation of these two groups yielded improved discrimination ability and predictive power regarding overall survival based on K‐M and time‐dependent ROC curve analyses (Figure 5e,f). Figure 5g reveals the 13 lncRNA expression profiles and the risk scores of 365 HCC patients with survival times via an heatmap and scatter plot, respectively. The vertical dotted line represents the optimal cutoff value of the risk score derived using the X‐tile approach mentioned previously. Univariate Cox regression analysis was subsequently conducted to screen potential indicators correlated with OS from 169 HCC patients with full clinical information. The results showed that the prognostic value of pathologic stage and tumor vascular invasion were statistically significant, similar to risk score. In the multivariate Cox regression analysis, vascular invasion was not associated with the prognosis of HCC patients. Thus, the risk score system derived from the expression levels of the 13 lncRNAs and pathologic stage were the only independent prognostic indicators of survival time for HCC patients (Figure 5h).

### 3.8 Correlations between lncRNAs and mRNAs

**Figure 6:**
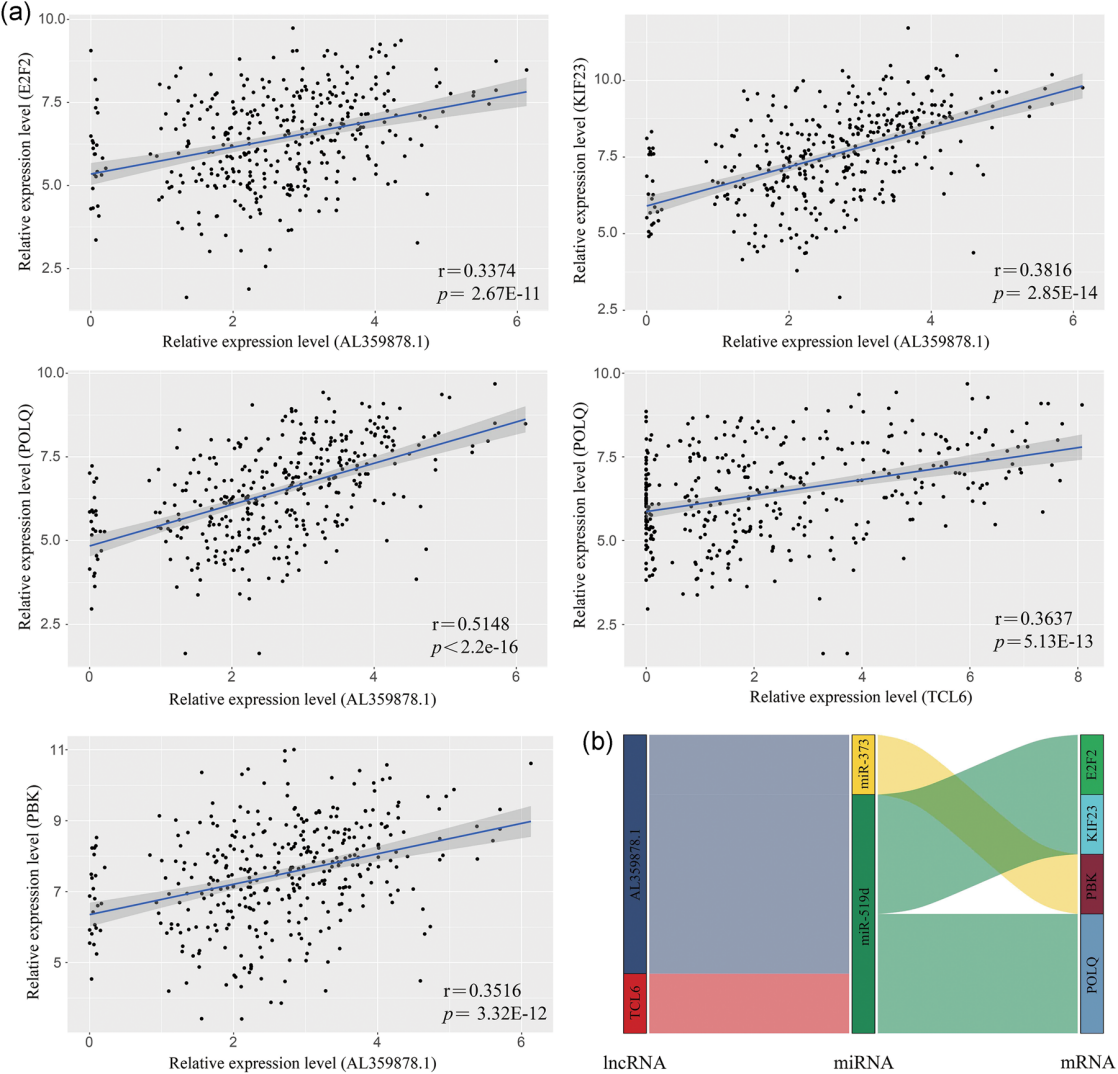
Correlation analysis of DEGs linear regression analysis between lncRNAs and mRNAs. LncRNAs versus protein coding genes as indicated (*n* = 370). The gray area around the blue line represent 95% confidence interval (a). Identified lncRNA–miRNA–mRNA axis are integrated into a module map (b). Left bar: lncRNA; middle bar: miRNA; right bar: mRNA. DEGs: differentially expressed genes; lncRNA: long noncoding RNA; miRNA: microRNA; mRNA: messenger RNA [Color figure can be viewed at wileyonlinelibrary.com]

According to the ceRNA mechanism theory, lncRNAs positively regulate mRNA expression by directly interacting with miRNAs. To verify this phenomenon in HCC, regression analysis of the 13 risk score‐related lncRNAs and 16 mRNAs that were significantly correlated with survival time was performed. Positive correlations were obtained for 13 lncRNA‐mRNA pairs (Table S5). Then, we investigated whether shared miRNAs existed between the lncRNAs and mRNAs. The results showed that miR‐519d is a key gene involved in multiple ceRNA pathways, including AL359878.1‐miR‐519d‐POLQ, AL359878.1‐miR‐519d‐KIF23, TCL6‐miR‐519d‐POLQ, and AL359878.1‐miR‐519d‐E2F2. In addition, the lncRNA AL359878.1 was positively correlated with mRNA *PBK* through another common miRNA, miR‐373 (Figure 6a,b).

## 4 DISCUSSION

HCC is the most common pathological type of liver cancer. The mortality rate of HCC patients ranks second among all cancers worldwide. In East Asia, Southeast Asia, Africa and southern Europe in particular, the incidences of liver cancer and associated mortality continue to rise (Bertuccio et al., 2017). Traditional surgical treatment can significantly improve the prognosis of some patients with HCC, but a large number of patients are intolerant to current treatments and experience recurrence and progression. Therefore, HCC‐related regulatory factors have become the focus of current research; such research is pivotal for achieving effective HCC treatment in the future. Furthermore, due to the development of high‐throughput sequencing technologies, lncRNAs have been found to play roles in transcriptional interference and to be indispensable for gene regulation, especially in ceRNA networks (Guttman & Rinn, 2012). Accumulating evidence has shown that ceRNA‐related genes greatly influence the occurrence, development and prognosis of most types of cancer (Schmitt & Chang, 2016).

The abnormal differentiation and development of liver cells lay the foundation for HCC genesis. To better understand the molecular mechanisms involved in the early occurrence of liver cancer, in an initial step, we identified shared aberrant lncRNAs, miRNAs, and mRNAs by comparing normal samples with HCC samples that showed varying degrees of differentiation, as determined using the TCGA database. After predicting the lncRNA–miRNA interactions and miRNA–mRNA interactions and excluding those lncRNAs that were distributed only in the nucleus, we constructed an HCC‐associated ceRNA regulatory network and performed GO and KEGG pathway enrichment analyses of the mRNAs in this network. K–M survival analysis revealed that a high percentage of genes was significantly correlated with overall survival in this network. Furthermore, we selected 13 of 15 survival‐related lncRNAs to calculate risk scores via multivariate Cox regression and lasso analysis. Finally, correlation analysis of lncRNAs and mRNAs was performed.

lncRNAs have multiple regulatory functions that vary based on the specificity of their subcellular localization. In the nucleus, lncRNAs can participate in chromatin interactions, transcriptional regulation and RNA processing, whereas within the cytoplasm, lncRNAs mainly function as regulatory factors of transcription products, translation and signaling pathways. Notably, the correlated lncRNAs, miRNAs, and mRNAs in the ceRNA regulatory network mainly interact with each other in the cytoplasm (Cao et al., 2018). To the best of our knowledge, our study is the first to consider not only the interactions of candidate genes but also the lncRNA distribution within the cell. To determine the expression localization of lncRNAs, we used ENSEMBL gene IDs to assess the lncRNAs from lncATLAS, which is an easy‐to‐use web‐based visualization tool. We did not incorporate the lncRNAs that existed only in the nucleus into the ceRNA network because the functioning of lncRNAs, miRNAs, and mRNAs as competitive endogenous RNAs mainly occurs in the cytoplasm.

The GO terms of the dysregulated mRNAs in the ceRNA network belonged predominantly to the following categories: “nucleoplasm,” “core promoter binding,” “transcription factor complex,” “spermatogenesis,” and “protein binding,” suggesting that HCC may be a metabolism‐related disease. “MicroRNAs in cancer,” “small cell lung cancer,” “cell cycle,” “gastric cancer,” “cushing syndrome,” “cellular senescence,” “p53 signaling pathway,” and “prostate cancer” were found to be the eight most enriched KEGG pathways in the KOBAS database analysis, indicating common abnormal signaling pathways in several cancer types, consistent with previous reports (X. L. Li, Zhou, Chen, & Chng, 2015; Mattioni et al., 2015). Strikingly, K–M survival analysis of the ceRNA‐correlated genes demonstrated that 15 of the 37 lncRNAs, 3 of the 10 miRNAs, and 16 of the 26 mRNAs had statistically significant influences on prognosis (*p*<0.01). When we evaluated significance at *p* < 0.05, 24 of the 37 lncRNAs, 5 of the 10 miRNAs, and 20 of the 26 mRNAs were strongly correlated with HCC patient prognosis (Table S5). Therefore, many genes significantly influence the overall survival time of HCC patients, demonstrating that an HCC‐associated ceRNA regulatory network can identify potential candidate biomarkers for predicting HCC patient prognosis.

In addition, in analyzing the 15 lncRNAs related to prognosis, lasso‐penalized Cox regression analysis excluded two lncRNAs, “DSCR8” and “AC006305.1.” Among the remaining lncRNAs, CRNDE is one of the best studied oncogenic lncRNAs. In most cancer types, such as glioma, pancreatic cancer, cervical cancer, colorectal carcinoma, GC, clear cell renal cell carcinoma, and hepatocellular carcinoma, CRNDE can facilitate the proliferation, migration, invasion and metastasis of cancer cells, leading to a poor prognosis for patients with the above cancer types (Ding et al., 2018; Hu, Du, Zhang, & Huang, 2017; Jiang et al., 2017; Meng, Li, Li, & Ma, 2017; Tang, Zheng, & Zhang, 2018; G. Wang, Pan, Zhang, Wei, & Wang, 2017; Zheng et al., 2016). Furthermore, high expression of the lncRNA PART1 indicates the probability of recurrence in non‐small‐cell lung cancer and hepatocellular carcinoma after curative resection (M. Li, Zhang, Zhang, Wang, & Lin, 2017; Lv et al., 2018). In addition, a previous study showed that PART1 can promote prostate cell proliferation and apoptosis by inhibiting TLR pathway activation (Sun, Geng, Li, Chen, & Zhao, 2018). LINC00462 is another confirmed oncogene that can enhance the progression of HCC and pancreatic cancer (Gong et al., 2017; Zhou, Guo, Sun, Zhang, & Zheng, 2018). CLLU1 mainly acts as a stable and inherent diagnostic biomarker of chronic lymphocytic leukemia and is significantly associated with poor clinical outcomes (Buhl et al., 2009; Gonzalez et al., 2013). In addition, as an independent prognostic factor, the good predictive power of the risk score prognostic model were proved via time‐dependent ROC curve analysis. Therefore, our ceRNA network identified not only a series of lncRNAs with unequivocal functions but also potential unexplored lncRNAs, including AL163952.1, AP002478.1, AC073352.1, LINC00221, and AL359878.1. AL359878.1 showed high positive correlations with the *POLQ, KIF23,* and *E2F2* mRNAs via miR‐519d and with the *PBK* mRNA via miR‐373.

Our study has some limitations. Due to the lack of other similar HCC‐associated lncRNA databases, external validation was not performed. Additionally, some exploratory experiments remain necessary to evaluate the functions of unreported lncRNAs.

## 5 CONCLUSIONS

In conclusion, we introduced a novel strategy for constructing an lncRNA–miRNA–mRNA ceRNA regulatory network according to the subcellular distributions of lncRNAs and interactions among genes. In addition to providing a comprehensive analysis network, this approach narrows the scope of research and enhances the prediction accuracy for target lncRNAs with great potential to serve as candidate biomarkers for the diagnosis, prognosis, and therapeutic targets of HCC patients.

## AUTHOR CONTRIBUTIONS

Y. B. and J. Y. L. conceived the study and performed the bioinformatics analyses. J. Z. L. and H. C. H. downloaded and organized the clinical and gene expression data. Z. S. L., D. X. W., X. Y., F. M. and Y. L. M. performed the statistical analyses. Y. B. wrote the manuscript. X. T. S. and H. T. Z. critically revised the article for essential intellectual content and administrative support. All authors read and approved the final version of the manuscript. All authors reviewed and revised the manuscript. H. T. Z. is the guarantor for this study.

## CONFLICT OF INTERESTS

The authors declare that there are no conflict of interests.

## Supporting information

Supporting information

Supporting information

Supporting information

Supporting information

Supporting information

Supporting information

## References

[jcp28522-bib-0001] Bertuccio, P. , Turati, F. , Carioli, G. , Rodriguez, T. , La Vecchia, C. , Malvezzi, M. , & Negri, E. (2017). Global trends and predictions in hepatocellular carcinoma mortality. Journal of Hepatology, 67(2), 302–309. 28336466 10.1016/j.jhep.2017.03.011

[jcp28522-bib-0002] Bray, F. , Ferlay, J. , Soerjomataram, I. , Siegel, R. L. , Torre, L. A. , & Jemal, A. (2018). Global cancer statistics 2018: GLOBOCAN estimates of incidence and mortality worldwide for 36 cancers in 185 countries. CA: A Cancer Journal for Clinicians, 68(6), 394–424. 30207593 10.3322/caac.21492

[jcp28522-bib-0003] Buhl, A. M. , Novotny, G. W. , Josefsson, P. , Nielsen, J. E. , Pedersen, L. B. , Geisler, C. , … Leffers, H. (2009). The CLLU1 expression level is a stable and inherent feature of the chronic lymphocytic leukemia clone. Leukemia, 23(6), 1182–1186. 19212335 10.1038/leu.2009.16 PMC5283720

[jcp28522-bib-0004] Camp, R. L. , Dolled‐Filhart, M. , & Rimm, D. L. (2004). X‐tile: A new bio‐informatics tool for biomarker assessment and outcome‐based cut‐point optimization. Clinical Cancer Research, 10(21), 7252–7259. 15534099 10.1158/1078-0432.CCR-04-0713

[jcp28522-bib-0005] Cao, Z. , Pan, X. , Yang, Y. , Huang, Y. , & Shen, H. B. (2018). The lncLocator: A subcellular localization predictor for long non‐coding RNAs based on a stacked ensemble classifier. Bioinformatics, 34(13), 2185–2194. 29462250 10.1093/bioinformatics/bty085

[jcp28522-bib-0006] Chou, C. H. , Chang, N. W. , Shrestha, S. , Hsu, S. D. , Lin, Y. L. , Lee, W. H. , … Huang, H. D. (2016). miRTarBase 2016: Updates to the experimentally validated miRNA‐target interactions database. Nucleic Acids Research, 44(D1), D239–D247. 26590260 10.1093/nar/gkv1258 PMC4702890

[jcp28522-bib-0007] Ding, C. , Han, F. , Xiang, H. , Xia, X. , Wang, Y. , Dou, M. , … Tian, P. (2018). LncRNA CRNDE is a biomarker for clinical progression and poor prognosis in clear cell renal cell carcinoma. Journal of Cellular Biochemistry, 119, 10406–10414. 30129055 10.1002/jcb.27389

[jcp28522-bib-0008] Fang, X. N. , Yin, M. , Li, H. , Liang, C. , Xu, C. , Yang, G. W. , & Zhang, H. X. (2018). Comprehensive analysis of competitive endogenous RNAs network associated with head and neck squamous cell carcinoma. Scientific Reports, 8(1), 10544. 30002503 10.1038/s41598-018-28957-y PMC6043529

[jcp28522-bib-0009] Fromm, B. , Billipp, T. , Peck, L. E. , Johansen, M. , Tarver, J. E. , King, B. L. , … Peterson, K. J. (2015). A uniform system for the annotation of vertebrate microRNA genes and the evolution of the human microRNAome. Annual Review of Genetics, 49, 213–242. 10.1146/annurev-genet-120213-092023 PMC4743252 26473382

[jcp28522-bib-0010] Gong, J. , Qi, X. , Zhang, Y. , Yu, Y. , Lin, X. , Li, H. , & Hu, Y. (2017). Long noncoding RNA linc00462 promotes hepatocellular carcinoma progression. Biomedicine and Pharmacotherapy, 93, 40–47. 28622593 10.1016/j.biopha.2017.06.004

[jcp28522-bib-0011] Gonzalez, D. , Else, M. , Wren, D. , Usai, M. , Buhl, A. M. , Parker, A. , … Catovsky, D. (2013). CLLU1 expression has prognostic value in chronic lymphocytic leukemia after first‐line therapy in younger patients and in those with mutated IGHV genes. Haematologica, 98(2), 274–278. 22899580 10.3324/haematol.2012.070201 PMC3561436

[jcp28522-bib-0012] Guttman, M. , & Rinn, J. L. (2012). Modular regulatory principles of large non‐coding RNAs. Nature, 482(7385), 339–346. 22337053 10.1038/nature10887 PMC4197003

[jcp28522-bib-0013] He, Y. , Meng, X. M. , Huang, C. , Wu, B. M. , Zhang, L. , Lv, X. W. , & Li, J. (2014). Long noncoding RNAs: Novel insights into hepatocelluar carcinoma. Cancer Letters, 344(1), 20–27. 24183851 10.1016/j.canlet.2013.10.021

[jcp28522-bib-0014] Hu, C. E. , Du, P. Z. , Zhang, H. D. , & Huang, G. J. (2017). Long noncoding RNA CRNDE promotes proliferation of gastric cancer cells by targeting miR‐145. Cellular Physiology and Biochemistry, 42(1), 13–21. 28490034 10.1159/000477107

[jcp28522-bib-0015] Huang Da, W. , Sherman, B. T. , & Lempicki, R. A. (2009). Systematic and integrative analysis of large gene lists using DAVID bioinformatics resources. Nature Protocols, 4(1), 44–57. 19131956 10.1038/nprot.2008.211

[jcp28522-bib-0016] Hutter, C. , & Zenklusen, J. C. (2018). The Cancer Genome atlas: Creating lasting value beyond its data. Cell, 173(2), 283–285. 29625045 10.1016/j.cell.2018.03.042

[jcp28522-bib-0017] Jeggari, A. , Marks, D. S. , & Larsson, E. (2012). miRcode: A map of putative microRNA target sites in the long non‐coding transcriptome. Bioinformatics, 28(15), 2062–2063. 22718787 10.1093/bioinformatics/bts344 PMC3400968

[jcp28522-bib-0018] Jiang, H. , Wang, Y. , Ai, M. , Wang, H. , Duan, Z. , Wang, H. , … Wang, S. (2017). Long noncoding RNA CRNDE stabilized by hnRNPUL2 accelerates cell proliferation and migration in colorectal carcinoma via activating Ras/MAPK signaling pathways. Cell Death & Disease, 8(6), e2862. 28594403 10.1038/cddis.2017.258 PMC5520914

[jcp28522-bib-0019] Kulik, L. , & El‐Serag, H. B. (2019). Epidemiology and management of hepatocellular carcinoma. Gastroenterology, 156(2), 477–491. e471 30367835 10.1053/j.gastro.2018.08.065 PMC6340716

[jcp28522-bib-0020] Li, C. , Li, R. , & Zhang, W. (2018). Progress in non‐invasive detection of liver fibrosis. Cancer Biology & Medicine, 15(2), 124–136. 29951337 10.20892/j.issn.2095-3941.2018.0018 PMC5994553

[jcp28522-bib-0021] Li, C. Y. , Liang, G. Y. , Yao, W. Z. , Sui, J. , Shen, X. , Zhang, Y. Q. , … Pu, Y. P. (2016). Integrated analysis of long non‐coding RNA competing interactions reveals the potential role in progression of human gastric cancer. International Journal of Oncology, 48(5), 1965–1976. 26935047 10.3892/ijo.2016.3407

[jcp28522-bib-0022] Li, M. , Zhang, W. , Zhang, S. , Wang, C. , & Lin, Y. (2017). PART1 expression is associated with poor prognosis and tumor recurrence in stage I‐III non‐small cell lung cancer. Journal of Cancer, 8(10), 1795–1800. 28819376 10.7150/jca.18848 PMC5556642

[jcp28522-bib-0023] Li, X. L. , Zhou, J. , Chen, Z. R. , & Chng, W. J. (2015). P53 mutations in colorectal cancer—Molecular pathogenesis and pharmacological reactivation. World Journal of Gastroenterology, 21(1), 84–93. 25574081 10.3748/wjg.v21.i1.84 PMC4284363

[jcp28522-bib-0024] Lv, Y. , Wei, W. , Huang, Z. , Chen, Z. , Fang, Y. , Pan, L. , … Xu, Z. (2018). Long non‐coding RNA expression profile can predict early recurrence in hepatocellular carcinoma after curative resection. Hepatology Research, 48, 1140–1148. 29924905 10.1111/hepr.13220

[jcp28522-bib-0025] Madar, V. , & Batista, S. (2016). FastLSU: A more practical approach for the Benjamini‐Hochberg FDR controlling procedure for huge‐scale testing problems. Bioinformatics, 32(11), 1716–1723. 26826716 10.1093/bioinformatics/btw029

[jcp28522-bib-0026] Mas‐Ponte, D. , Carlevaro‐Fita, J. , Palumbo, E. , Hermoso Pulido, T. , Guigo, R. , & Johnson, R. (2017). LncATLAS database for subcellular localization of long noncoding RNAs. RNA, 23(7), 1080–1087. 28386015 10.1261/rna.060814.117 PMC5473142

[jcp28522-bib-0027] Mattioni, M. , Soddu, S. , Prodosmo, A. , Visca, P. , Conti, S. , Alessandrini, G. , … Strigari, L. (2015). Prognostic role of serum p53 antibodies in lung cancer. BMC Cancer, 15, 148. 25884692 10.1186/s12885-015-1174-4 PMC4374590

[jcp28522-bib-0028] Meng, Y. , Li, Q. , Li, L. , & Ma, R. (2017). The long non‐coding RNA CRNDE promotes cervical cancer cell growth and metastasis. Biological Chemistry, 399(1), 93–100. 29194035 10.1515/hsz-2017-0199

[jcp28522-bib-0029] Quinn, J. J. , & Chang, H. Y. (2016). Unique features of long non‐coding RNA biogenesis and function. Nature Reviews Genetics, 17(1), 47–62. 10.1038/nrg.2015.10 26666209

[jcp28522-bib-0030] Robinson, M. D. , McCarthy, D. J. , & Smyth, G. K. (2010). edgeR: A Bioconductor package for differential expression analysis of digital gene expression data. Bioinformatics, 26(1), 139–140. 19910308 10.1093/bioinformatics/btp616 PMC2796818

[jcp28522-bib-0031] Salmena, L. , Poliseno, L. , Tay, Y. , Kats, L. , & Pandolfi, P. P. (2011). A ceRNA hypothesis: The Rosetta stone of a hidden RNA language? Cell, 146(3), 353–358. 21802130 10.1016/j.cell.2011.07.014 PMC3235919

[jcp28522-bib-0032] Schmitt, A. M. , & Chang, H. Y. (2016). Long noncoding RNAs in cancer pathways. Cancer Cell, 29(4), 452–463. 27070700 10.1016/j.ccell.2016.03.010 PMC4831138

[jcp28522-bib-0033] Shannon, P. , Markiel, A. , Ozier, O. , Baliga, N. S. , Wang, J. T. , Ramage, D. , … Ideker, T. (2003). Cytoscape: A software environment for integrated models of biomolecular interaction networks. Genome Research, 13(11), 2498–2504. 14597658 10.1101/gr.1239303 PMC403769

[jcp28522-bib-0034] Siegel, R. L. , Miller, K. D. , & Jemal, A. (2018). Cancer statistics, 2018. CA: A Cancer Journal for Clinicians, 68(1), 7–30. 29313949 10.3322/caac.21442

[jcp28522-bib-0035] Sun, M. , Geng, D. , Li, S. , Chen, Z. , & Zhao, W. (2018). LncRNA PART1 modulates toll‐like receptor pathways to influence cell proliferation and apoptosis in prostate cancer cells. Biological Chemistry, 399(4), 387–395. 29261512 10.1515/hsz-2017-0255

[jcp28522-bib-0036] Tang, Q. , Zheng, X. , & Zhang, J. (2018). Long non‐coding RNA CRNDE promotes heptaocellular carcinoma cell proliferation by regulating PI3K/Akt /beta‐catenin signaling. Biomedicine & Pharmacotherapy, 103, 1187–1193. 29864897 10.1016/j.biopha.2018.04.128

[jcp28522-bib-0037] Wang, G. , Pan, J. , Zhang, L. , Wei, Y. , & Wang, C. (2017). Long non‐coding RNA CRNDE sponges miR‐384 to promote proliferation and metastasis of pancreatic cancer cells through upregulating IRS1. Cell Proliferation, 50(6), e12389. 10.1111/cpr.12389 PMC6529119 28940804

[jcp28522-bib-0038] Wang, H. , Huo, X. , Yang, X. R. , He, J. , Cheng, L. , Wang, N. , … Qin, W. (2017). STAT3‐mediated upregulation of lncRNA HOXD‐AS1 as a ceRNA facilitates liver cancer metastasis by regulating SOX4. Molecular Cancer, 16(1), 136. 28810927 10.1186/s12943-017-0680-1 PMC5558651

[jcp28522-bib-0039] Wang, Y. , Sun, L. , Wang, L. , Liu, Z. , Li, Q. , Yao, B. , … Liu, Q. (2018). Long non‐coding RNA DSCR8 acts as a molecular sponge for miR‐485‐5p to activate Wnt/beta‐catenin signal pathway in hepatocellular carcinoma. Cell Death & Disease, 9(9), 851. 30154476 10.1038/s41419-018-0937-7 PMC6113322

[jcp28522-bib-0040] Wong, N. , & Wang, X. (2015). miRDB: An online resource for microRNA target prediction and functional annotations. Nucleic Acids Research, 43(Database issue), D146–D152. 25378301 10.1093/nar/gku1104 PMC4383922

[jcp28522-bib-0041] Xie, C. , Mao, X. , Huang, J. , Ding, Y. , Wu, J. , Dong, S. , … Wei, L. (2011). KOBAS 2.0: A web server for annotation and identification of enriched pathways and diseases. Nucleic Acids Research, 39(Web Server issue), W316–W322. 21715386 10.1093/nar/gkr483 PMC3125809

[jcp28522-bib-0042] Xu, S. , Sui, J. , Yang, S. , Liu, Y. , Wang, Y. , & Liang, G. (2018). Integrative analysis of competing endogenous RNA network focusing on long noncoding RNA associated with progression of cutaneous melanoma. Cancer Medicine, 7(4), 1019–1029. 29522273 10.1002/cam4.1315 PMC5911588

[jcp28522-bib-0043] Zheng, J. , Liu, X. , Wang, P. , Xue, Y. , Ma, J. , Qu, C. , & Liu, Y. (2016). CRNDE promotes malignant progression of glioma by attenuating miR‐384/PIWIL4/STAT3 axis. Molecular Therapy, 24(7), 1199–1215. 27058823 10.1038/mt.2016.71 PMC5088760

[jcp28522-bib-0044] Zhou, B. , Guo, W. , Sun, C. , Zhang, B. , & Zheng, F. (2018). Linc00462 promotes pancreatic cancer invasiveness through the miR‐665/TGFBR1‐TGFBR2/SMAD2/3 pathway. Cell Death & Disease, 9(6), 706. 29899418 10.1038/s41419-018-0724-5 PMC5999603

