# Supplementary material for: Comprehensive analysis of a ceRNA network reveals potential prognostic cytoplasmic lncRNAs involved in HCC progression
Source: J Cell Physiol. 2019 Mar 27;234(10):18837–48. doi: 10.1002/jcp.28522 (PMC6618076; doi:10.1002/jcp.28522)
Supplement: Supplementary file 1 — Supporting information [file JCP-234-18837-s001.docx]

Tables

Table S1

| **lncRNA** | **Gene ID** | **Log FC** | ***P*-value** | **miRNA** |
| --- | --- | --- | --- | --- |
| AC006305.1 | ENSG00000206129 | 2.973629989 | 3.28377E-07 | miR-137, miR-424, miR-519d, miR-182, miR-216a |
| AC009065.1 | ENSG00000259780 | 2.073707267 | 7.15319E-07 | miR-372, miR-373 |
| AC016773.1 | ENSG00000270195 | 2.333996304 | 7.16608E-26 | miR-424, miR-216a, miR-217 |
| AC024563.1 | ENSG00000268981 | 3.339548743 | 1.62467E-06 | miR-183, miR-217 |
| AC040173.1 | ENSG00000263257 | 2.803843085 | 1.12358E-06 | miR-96, miR-519d, miR-182, miR-183, miR-217 |
| AC061975.6 | ENSG00000266830 | 5.132765879 | 1.19418E-07 | miR-372, miR-373, miR-519d |
| AC073352.1 | ENSG00000272662 | 2.324520683 | 7.7044E-18 | miR-96, miR-182 |
| AC087392.1 | ENSG00000262003 | -2.588928447 | 1.49629E-18 | miR-372, miR-373, miR-424, miR-519d, miR-216a |
| AL033381.1 | ENSG00000176515 | 2.022678256 | 0.000273544 | miR-424 |
| AL163952.1 | ENSG00000259868 | 2.903624898 | 3.87248E-08 | miR-96, miR-182, miR-205, miR-216b |
| AL357153.1 | ENSG00000245466 | 2.759022703 | 1.12025E-06 | miR-183, miR-217 |
| AL359878.1 | ENSG00000205740 | 2.228738261 | 1.96592E-17 | miR-372, miR-373, miR-519d, miR-216a |
| AL512652.1 | ENSG00000275485 | 2.636231727 | 5.14665E-18 | miR-519d, miR-205 |
| AL713998.1 | ENSG00000227706 | 4.796260091 | 2.59231E-05 | miR-137 |
| AP002478.1 | ENSG00000266401 | 5.069727076 | 1.97384E-11 | miR-372, miR-373, miR-424, miR-519d, miR-182, miR-184, miR-205, miR-216a |
| BPESC1 | ENSG00000232416 | 3.465939992 | 3.43909E-10 | miR-424, miR-205, miR-216a, miR-216b |
| C10orf91 | ENSG00000180066 | 4.405958355 | 8.79368E-09 | miR-372, miR-373 |
| CLDN10-AS1 | ENSG00000223392 | 4.156648241 | 1.18693E-05 | miR-137 |
| CLLU1 | ENSG00000257127 | 3.859265273 | 1.55322E-05 | miR-372, miR-373, miR-137, miR-424, miR-519d, miR-205, miR-216a, miR-216b, miR-217 |
| CRNDE | ENSG00000245694 | 3.182416908 | 1.38735E-24 | miR-183, miR-205, miR-216b, miR-217 |
| DLX6-AS1 | ENSG00000231764 | 5.275300959 | 5.97982E-11 | miR-372, miR-373, miR-424, miR-519d, miR-216a, miR-216b |
| DSCR10 | ENSG00000233316 | 2.821378658 | 0.000192184 | miR-424 |
| DSCR4 | ENSG00000184029 | 6.857021227 | 8.66219E-10 | miR-216b |
| DSCR8 | ENSG00000198054 | 7.431623051 | 2.87018E-14 | miR-137, miR-205 |
| ERVMER61-1 | ENSG00000230426 | 4.419993177 | 4.74546E-07 | miR-96, miR-182, miR-205 |
| FAM87A | ENSG00000182366 | 2.996535582 | 1.09059E-09 | miR-96, miR-424, miR-519d, miR-205, miR-216b |
| GDNF-AS1 | ENSG00000248587 | 2.32799598 | 9.7438E-07 | miR-424, miR-216a |
| HOTAIR | ENSG00000228630 | 3.134566963 | 0.000173741 | miR-519d, miR-216a, miR-216b, miR-217 |
| HOTTIP | ENSG00000243766 | 7.124530903 | 3.10529E-29 | miR-372, miR-373, miR-137, miR-424, miR-519d, miR-184, miR-205, miR-216a |
| HTR2A-AS1 | ENSG00000224517 | -2.375753336 | 6.35173E-26 | miR-137 |
| LINC00114 | ENSG00000223806 | 2.774847311 | 2.89816E-08 | miR-96, miR-182, miR-216a |
| LINC00160 | ENSG00000230978 | 3.16596866 | 6.44277E-06 | miR-424 |
| LINC00200 | ENSG00000229205 | 3.843854339 | 3.09304E-05 | miR-424, miR-519d, miR-183, miR-216a, miR-216b, miR-217 |
| LINC00221 | ENSG00000270816 | 7.584568655 | 3.21139E-14 | miR-372, miR-373, miR-96, miR-519d, miR-182,  miR-217 |
| LINC00355 | ENSG00000227674 | 7.179096458 | 2.93346E-20 | miR-424 |
| LINC00462 | ENSG00000233610 | 2.634333579 | 8.84863E-05 | miR-372, miR-373, miR-519d |
| LINC00473 | ENSG00000223414 | 2.643151077 | 0.000149447 | miR-424 |
| LINC00485 | ENSG00000258169 | 2.228058535 | 1.23261E-09 | miR-372, miR-373, miR-424, miR-205, miR-216a |
| LINC00488 | ENSG00000214381 | 3.429333447 | 6.90417E-08 | miR-96, miR-205, miR-216a, miR-216b |
| LINC00491 | ENSG00000250682 | 5.575995182 | 2.27399E-11 | miR-184, miR-216b |
| LINC00494 | ENSG00000235621 | 3.218272355 | 2.31361E-07 | miR-372, miR-373, miR-182, miR-217 |
| LINC00501 | ENSG00000203645 | 2.774383308 | 1.39851E-07 | miR-183 |
| LINC00519 | ENSG00000258955 | 2.916371385 | 1.2111E-08 | miR-216a |
| MIR137HG | ENSG00000225206 | 3.202950584 | 2.79E-05 | miR-182, miR-217 |
| MYCNOS | ENSG00000233718 | 2.570058662 | 1.68875E-07 | miR-183, miR-205, miR-217 |
| NOVA1-AS1 | ENSG00000257842 | 5.693545077 | 5.1962E-14 | miR-216a, miR-216b, miR-217 |
| PART1 | ENSG00000152931 | 6.905733259 | 1.5281E-11 | miR-424, miR-205 |
| RMST | ENSG00000255794 | 3.521329643 | 1.22919E-11 | miR-96, miR-137, miR-424, miR-519d, miR-182, miR-205 |
| SACS-AS1 | ENSG00000229558 | 6.426658137 | 3.08477E-08 | miR-372, miR-373, miR-205 |
| SFTA1P | ENSG00000225383 | 5.417701568 | 8.56793E-47 | miR-424, miR-182, miR-216a, miR-216b |
| TCL6 | ENSG00000187621 | 2.587811877 | 4.52891E-08 | miR-372, miR-373, miR-96, miR-137, miR-424, miR-519d, miR-182, miR-183, miR-205, miR-216a, miR-216b, miR-217 |
| TDRG1 | ENSG00000204091 | 4.628259032 | 1.47598E-08 | miR-519d |
| ZNF385D-AS1 | ENSG00000225542 | 5.349981151 | 1.2455E-09 | miR-205 |
