# Supplementary material for: Comprehensive analysis of a ceRNA network reveals potential prognostic cytoplasmic lncRNAs involved in HCC progression
Source: J Cell Physiol. 2019 Mar 27;234(10):18837–48. doi: 10.1002/jcp.28522 (PMC6618076; doi:10.1002/jcp.28522)
Supplement: Supplementary file 2 — Supporting information [file JCP-234-18837-s002.docx]

Table S2

| **miRNA** | **mRNA** |
| --- | --- |
| miR-137 | PTGS2 |
| miR-182 | HOXA9, NPTX1 |
| miR-183 | CCNB1 |
| miR-205 | ACSL4 |
| miR-217 | DACH1, EZH2 |
| miR-372 | SLC7A11, ELAVL2 |
| miR-373 | PBK, ELAVL2, SLC7A11 |
| miR-424 | HOXA3, HOXA10, CCNE1, E2F7, CLSPN, KIF23, CBX2, GNAL, CEP55, AXIN2 |
| miR-519d | ACSL4, E2F1, ELAVL2, RRM2, NETO2, POLQ, E2F2, KIF23 |
| miR-96 | PROK2 |
