# Supplementary material for: Comprehensive analysis of a ceRNA network reveals potential prognostic cytoplasmic lncRNAs involved in HCC progression
Source: J Cell Physiol. 2019 Mar 27;234(10):18837–48. doi: 10.1002/jcp.28522 (PMC6618076; doi:10.1002/jcp.28522)
Supplement: Supplementary file 3 — Supporting information [file JCP-234-18837-s003.docx]

Table S3

| **LncRNA name** | **Location** |
| --- | --- |
| C10orf91 | Both |
| TDRG1 | Both |
| SFTA1P | Both |
| LINC00355 | Both |
| HOTAIR | Both |
| MIR137HG | Both |
| DLX6-AS1 | Both |
| CRNDE | Both |
| RMST | Both |
| PART1 | Cytoplasmic |
| LINC00221 | Cytoplasmic |
| LINC00462 | Cytoplasmic |
| MYCNOS | Cytoplasmic |
| LINC00485 | Cytoplasmic |
| LINC00519 | Cytoplasmic |
| AC009065.1 | No data |
| AL357153.1 | No data |
| AL033381.1 | No data |
| AP002478.1 | No data |
| AC024563.1 | No data |
| DSCR4 | No data |
| TCL6 | No data |
| AC087392.1 | No data |
| DSCR8 | No data |
| AC061975.6 | No data |
| AL512652.1 | No data |
| CLLU1 | No data |
| AL359878.1 | No data |
| AC006305.1 | No data |
| AL163952.1 | No data |
| AC016773.1 | No data |
| LINC00114 | No data |
| HTR2A-AS1 | No data |
| ZNF385D-AS1 | No data |
| AL713998.1 | No data |
| SACS-AS1 | No data |
| BPESC1 | No data |
| AC073352.1 | No data |
| LINC00491 | No data |
| AC040173.1 | No data |
| FAM87A | Nuclear |
| LINC00501 | Nuclear |
| LINC00488 | Nuclear |
| CLDN10-AS1 | Nuclear |
| LINC00473 | Nuclear |
| LINC00200 | Nuclear |
| ERVMER61-1 | Nuclear |
| LINC00160 | Nuclear |
| DSCR10 | Nuclear |
| LINC00494 | Nuclear |
| HOTTIP | Nuclear |
| GDNF-AS1 | Nuclear |
| NOVA1-AS1 | Nuclear |
