# Supplementary material for: Comprehensive analysis of a ceRNA network reveals potential prognostic cytoplasmic lncRNAs involved in HCC progression
Source: J Cell Physiol. 2019 Mar 27;234(10):18837–48. doi: 10.1002/jcp.28522 (PMC6618076; doi:10.1002/jcp.28522)
Supplement: Supplementary file 4 — Supporting information [file JCP-234-18837-s004.docx]

Table S4

| **Category** | **ID** | **Term** | **Target Genes** | ***P*-Value** |
| --- | --- | --- | --- | --- |
| CC | GO:0005654 | nucleoplasm | KIF23, E2F1, CLSPN, E2F2, E2F7, EZH2, CBX2, ELAVL2, CCNB1, CCNE1, HOXA3, RRM2, POLQ, AXIN2 | 2.03E-05 |
| MF | GO:0001047 | core promoter binding | E2F1, E2F2, E2F7, EZH2 | 1.13E-04 |
| CC | GO:0005667 | transcription factor complex | E2F2, E2F7, HOXA10, HOXA9, DACH1 | 1.30E-04 |
| BP | GO:0007283 | spermatogenesis | CCNB1, E2F1, PROK2, HOXA10, HOXA9 | 0.0023498 |
| MF | GO:0005515 | protein binding | E2F1, KIF23, CLSPN, E2F2, PTGS2, E2F7, EZH2, ELAVL2, CBX2, PBK, CEP55, DACH1, SLC7A11, CCNB1, CCNE1, HOXA3, RRM2, HOXA10, HOXA9, AXIN2, POLQ | 0.0037479 |
| BP | GO:0006977 | DNA damage response, signal transduction by p53 class mediator resulting in cell cycle arrest | CCNB1, E2F1, E2F7 | 0.0038098 |
| BP | GO:0071930 | negative regulation of transcription involved in G1/S transition of mitotic cell cycle | E2F1, E2F7 | 0.00446 |
| BP | GO:0006355 | regulation of transcription, DNA-templated | E2F1, E2F2, HOXA3, EZH2, HOXA10, HOXA9, ELAVL2, AXIN2 | 0.0051769 |
| CC | GO:0005634 | nucleus | KIF23, E2F1, PTGS2, E2F7, EZH2, CBX2, PBK, DACH1, CCNB1, CCNE1, HOXA3, RRM2, HOXA10, HOXA9, AXIN2 | 0.0054231 |
| BP | GO:1990086 | lens fiber cell apoptotic process | E2F1, E2F2 | 0.0059425 |
| BP | GO:0009952 | anterior/posterior pattern specification | HOXA3, HOXA10, HOXA9 | 0.0062632 |
| BP | GO:0071456 | cellular response to hypoxia | CCNB1, E2F1, PTGS2 | 0.0089076 |
| MF | GO:0003677 | DNA binding | E2F1, CLSPN, E2F2, E2F7, EZH2, CBX2, DACH1, POLQ | 0.0090092 |
