# Supplementary material for: Comprehensive analysis of a ceRNA network reveals potential prognostic cytoplasmic lncRNAs involved in HCC progression
Source: J Cell Physiol. 2019 Mar 27;234(10):18837–48. doi: 10.1002/jcp.28522 (PMC6618076; doi:10.1002/jcp.28522)
Supplement: Supplementary file 5 — Supporting information [file JCP-234-18837-s005.docx]

Table S5

| **lncRNA** | **Level** | **Label** | **N** | ***P*-Value** |
| --- | --- | --- | --- | --- |
| MIR137HG | high | >2.662748025 | 39 | 2.04E-07 |
| MIR137HG | low | <=2.662748025 | 331 | 2.04E-07 |
| AP002478.1 | high | >1.290898532 | 135 | 3.25E-06 |
| AP002478.1 | low | <=1.290898532 | 235 | 3.25E-06 |
| LINC00462 | high | >1.499482236 | 43 | 1.73E-05 |
| LINC00462 | low | <=1.499482236 | 327 | 1.73E-05 |
| AL163952.1 | high | >9.659420867 | 76 | 2.82E-05 |
| AL163952.1 | low | <=9.659420867 | 294 | 2.82E-05 |
| AL359878.1 | high | >8.929393889 | 106 | 2.92E-05 |
| AL359878.1 | low | <=8.929393889 | 264 | 2.92E-05 |
| C10orf91 | high | >5.149475245 | 72 | 3.74E-05 |
| C10orf91 | low | <=5.149475245 | 298 | 3.74E-05 |
| HTR2A-AS1 | high | >0.057498654 | 195 | 0.000101 |
| HTR2A-AS1 | low | <=0.057498654 | 175 | 0.000101 |
| TCL6 | high | >10.77944822 | 106 | 0.000206 |
| TCL6 | low | <=10.77944822 | 264 | 0.000206 |
| LINC00221 | high | >383.8241356 | 62 | 0.000316 |
| LINC00221 | low | <=383.8241356 | 308 | 0.000316 |
| AC073352.1 | high | >3.796461806 | 163 | 0.000886 |
| AC073352.1 | low | <=3.796461806 | 207 | 0.000886 |
| AC006305.1 | high | >32.5261647 | 52 | 0.00112 |
| AC006305.1 | low | <=32.5261647 | 318 | 0.00112 |
| PART1 | high | >60.22527263 | 41 | 0.001764 |
| PART1 | low | <=60.22527263 | 329 | 0.001764 |
| CRNDE | high | >203.1371709 | 111 | 0.003155 |
| CRNDE | low | <=203.1371709 | 259 | 0.003155 |
| CLLU1 | high | >0.032158915 | 195 | 0.003767 |
| CLLU1 | low | <=0.032158915 | 175 | 0.003767 |
| DSCR8 | high | >58.99114996 | 59 | 0.005863 |
| DSCR8 | low | <=58.99114996 | 311 | 0.005863 |
| DLX6-AS1 | high | >54.19006627 | 43 | 0.011047 |
| DLX6-AS1 | low | <=54.19006627 | 327 | 0.011047 |
| AC016773.1 | high | >24.05650101 | 83 | 0.011073 |
| AC016773.1 | low | <=24.05650101 | 287 | 0.011073 |
| SACS-AS1 | high | >2.823747876 | 39 | 0.011277 |
| SACS-AS1 | low | <=2.823747876 | 331 | 0.011277 |
| MYCNOS | high | >16.79279197 | 37 | 0.012549 |
| MYCNOS | low | <=16.79279197 | 333 | 0.012549 |
| LINC00114 | high | >0.020375002 | 291 | 0.022836 |
| LINC00114 | low | <=0.020375002 | 79 | 0.022836 |
| BPESC1 | high | >0.50665674 | 173 | 0.033274 |
| BPESC1 | low | <=0.50665674 | 197 | 0.033274 |
| AL512652.1 | high | >3.947769929 | 103 | 0.037824 |
| AL512652.1 | low | <=3.947769929 | 267 | 0.037824 |
| AC087392.1 | high | >26.30414979 | 86 | 0.040631 |
| AC087392.1 | low | <=26.30414979 | 284 | 0.040631 |
| TDRG1 | high | >2.035233944 | 43 | 0.049283 |
| TDRG1 | low | <=2.035233944 | 327 | 0.049283 |
|  |  |  |  |  |
| **miRNA** | **Level** | **Label** | **N** | ***P*-Value** |
| hsa-mir-137 | high | >7.38809307099617 | 40 | 0.000246 |
| hsa-mir-137 | low | <=7.38809307099617 | 326 | 0.000246 |
| hsa-mir-372 | high | >10.4672548201992 | 42 | 0.001175 |
| hsa-mir-372 | low | <=10.4672548201992 | 324 | 0.001175 |
| hsa-mir-182 | high | >63488.383146089 | 85 | 0.008855 |
| hsa-mir-182 | low | <=63488.383146089 | 281 | 0.008855 |
| hsa-mir-183 | high | >44258.4141951885 | 38 | 0.029292 |
| hsa-mir-183 | low | <=44258.4141951885 | 328 | 0.029292 |
| hsa-mir-373 | high | >2.42133924441754 | 58 | 0.037781 |
| hsa-mir-373 | low | <=2.42133924441754 | 308 | 0.037781 |
|  |  |  |  |  |
| **mRNA** | **Level** | **Label** | **N** | ***P*-Value** |
| CBX2 | high | >252.32737 | 104 | 1.96E-09 |
| CBX2 | low | <=252.32737 | 266 | 1.96E-09 |
| CEP55 | high | >207.8794967 | 111 | 2.25E-09 |
| CEP55 | low | <=207.8794967 | 259 | 2.25E-09 |
| SLC7A11 | high | >442.8609561 | 83 | 2.52E-07 |
| SLC7A11 | low | <=442.8609561 | 287 | 2.52E-07 |
| CLSPN | high | >53.81514581 | 165 | 2.86E-06 |
| CLSPN | low | <=53.81514581 | 205 | 2.86E-06 |
| EZH2 | high | >415.3998202 | 209 | 4.45E-06 |
| EZH2 | low | <=415.3998202 | 161 | 4.45E-06 |
| E2F2 | high | >75.82827256 | 205 | 5.22E-06 |
| E2F2 | low | <=75.82827256 | 165 | 5.22E-06 |
| RRM2 | high | >1437.665778 | 134 | 2.47E-05 |
| RRM2 | low | <=1437.665778 | 236 | 2.47E-05 |
| PBK | high | >405.2873319 | 79 | 2.51E-05 |
| PBK | low | <=405.2873319 | 291 | 2.51E-05 |
| CCNB1 | high | >456.7359351 | 251 | 3.77E-05 |
| CCNB1 | low | <=456.7359351 | 119 | 3.77E-05 |
| E2F1 | high | >417.6029921 | 232 | 7.1E-05 |
| E2F1 | low | <=417.6029921 | 138 | 7.1E-05 |
| POLQ | high | >131.2839671 | 133 | 7.69E-05 |
| POLQ | low | <=131.2839671 | 237 | 7.69E-05 |
| KIF23 | high | >256.6844859 | 154 | 0.000148 |
| KIF23 | low | <=256.6844859 | 216 | 0.000148 |
| E2F7 | high | >46.3890198 | 200 | 0.000156 |
| E2F7 | low | <=46.3890198 | 170 | 0.000156 |
| PROK2 | high | >0.04137228 | 280 | 0.000638 |
| PROK2 | low | <=0.04137228 | 90 | 0.000638 |
| CCNE1 | high | >123.1950811 | 166 | 0.000669 |
| CCNE1 | low | <=123.1950811 | 204 | 0.000669 |
| NPTX1 | high | >17.17209287 | 50 | 0.003887 |
| NPTX1 | low | <=17.17209287 | 320 | 0.003887 |
| GNAL | high | >63.66481592 | 287 | 0.015705 |
| GNAL | low | <=63.66481592 | 83 | 0.015705 |
| HOXA9 | high | >9.374472698 | 56 | 0.028777 |
| HOXA9 | low | <=9.374472698 | 314 | 0.028777 |
| AXIN2 | high | >61.48667463 | 244 | 0.033938 |
| AXIN2 | low | <=61.48667463 | 126 | 0.033938 |
| HOXA3 | high | >100.9751749 | 126 | 0.044752 |
| HOXA3 | low | <=100.9751749 | 244 | 0.044752 |
