# Supplementary material for: Comprehensive analysis of a ceRNA network reveals potential prognostic cytoplasmic lncRNAs involved in HCC progression
Source: J Cell Physiol. 2019 Mar 27;234(10):18837–48. doi: 10.1002/jcp.28522 (PMC6618076; doi:10.1002/jcp.28522)
Supplement: Supplementary file 6 — Supporting information [file JCP-234-18837-s006.docx]

Table S6

| **lncRNA** | **miRNA** | **mRNA** | ***P*-Value** | **R** |
| --- | --- | --- | --- | --- |
| AL359878.1 | miR-519d | POLQ | < 2.2e-16 | 0.5148158 |
| AL359878.1 | miR-519d | KIF23 | < 2.2e-16 | 0.5124544 |
| AL359878.1 | NA | CLSPN | < 2.2e-16 | 0.494009 |
| AL359878.1 | NA | CEP55 | < 2.2e-16 | 0.4638198 |
| AC073352.1 | NA | CCNB1 | 1.56E-15 | 0.3984939 |
| MIR137HG | NA | CEP55 | 3.05E-15 | 0.3946911 |
| MIR137HG | NA | KIF23 | 2.85E-14 | 0.3816003 |
| AL359878.1 | NA | EZH2 | 2.42E-13 | 0.3684837 |
| TCL6 | miR-519d | POLQ | 5.13E-13 | 0.363734 |
| AL359878.1 | miR-373 | PBK | 3.32E-12 | 0.351581 |
| MIR137HG | NA | CCNB1 | 3.61E-12 | 0.3510262 |
| AL359878.1 | miR-519d | E2F2 | 2.67E-11 | 0.3373556 |
| AL359878.1 | NA | CCNB1 | 1.11E-10 | 0.3271825 |
